# The Flavonoid Quercetin Reverses Pulmonary Hypertension in Rats

**DOI:** 10.1371/journal.pone.0114492

**Published:** 2014-12-02

**Authors:** Daniel Morales-Cano, Carmen Menendez, Enrique Moreno, Javier Moral-Sanz, Bianca Barreira, Pilar Galindo, Rachele Pandolfi, Rosario Jimenez, Laura Moreno, Angel Cogolludo, Juan Duarte, Francisco Perez-Vizcaino

**Affiliations:** 1 Department of Pharmacology, School of Medicine, University Complutense of Madrid, Madrid, Spain; 2 Ciber Enfermedades Respiratorias (CIBERES), Madrid, Spain; 3 Faculty of Health Sciences, Universidad Autónoma de Chile, Santiago, Chile; 4 Department of Pharmacology, School of Pharmacy, University of Granada, Granada, Spain; Texas A& M University Health Science Center, United States of America

## Abstract

Quercetin is a dietary flavonoid which exerts vasodilator, antiplatelet and antiproliferative effects and reduces blood pressure, oxidative status and end-organ damage in humans and animal models of systemic hypertension. We hypothesized that oral quercetin treatment might be protective in a rat model of pulmonary arterial hypertension. Three weeks after injection of monocrotaline, quercetin (10 mg/kg/d per os) or vehicle was administered for 10 days to adult Wistar rats. Quercetin significantly reduced mortality. In surviving animals, quercetin decreased pulmonary arterial pressure, right ventricular hypertrophy and muscularization of small pulmonary arteries. Classic biomarkers of pulmonary arterial hypertension such as the downregulated expression of lung BMPR2, Kv1.5, Kv2.1, upregulated survivin, endothelial dysfunction and hyperresponsiveness to 5-HT were unaffected by quercetin. Quercetin significantly restored the decrease in Kv currents, the upregulation of 5-HT_2A_ receptors and reduced the Akt and S6 phosphorylation. In vitro, quercetin induced pulmonary artery vasodilator effects, inhibited pulmonary artery smooth muscle cell proliferation and induced apoptosis. In conclusion, quercetin is partially protective in this rat model of PAH. It delayed mortality by lowering PAP, RVH and vascular remodeling. Quercetin exerted effective vasodilator effects in isolated PA, inhibited cell proliferation and induced apoptosis in PASMCs. These effects were associated with decreased 5-HT_2A_ receptor expression and Akt and S6 phosphorylation and partially restored Kv currents. Therefore, quercetin could be useful in the treatment of PAH.

## Introduction

Pulmonary arterial hypertension (PAH) is a rare disease characterized by elevated pulmonary arterial pressure (PAP) due to increased vasoconstriction, remodeling of the pulmonary microvasculature and thrombosis, leading to right ventricular hypertrophy (RVH) and premature death [1]. PAH exhibits a complex pathophysiology, unlikely to be explained by a single factor [2], [3]. Mutations in the bone morphogenetic protein receptor type 2 (BMPR2) are responsible for many heritable forms of PAH and downregulation of its expression underlie many idiopathic and secondary forms of PAH [4], [5]. BMPR2 dysfunction leads to increased transforming growth factor-β (TGF-β) signaling [6] leading to activation of proliferative pathways including the mitogen activated protein kinases (MAPKs) pathway, the phosphatidylinositide 3-kinases, serine/threonine kinase Akt and the mammalian target of rapamycin (PI3K/Akt/mTOR) pathway and the antiapoptotic protein survivin. Inactivation, downregulation or gene polymorphisms of voltage-gated potassium channels (K_V_) [7], hyperresponsiveness to 5-HT [8] and loss of NO bioavailability and the subsequent endothelial dysfunction have also been implicated in the pathophysiology of PAH [9]. Although no cure exists for PAH, the understanding of the pathophysiological mechanisms has led to the development of therapies which improve symptoms and slow the progression of the disease [10].

Quercetin is a natural flavonoid regularly consumed in the diet in the form of fruits, vegetables, nuts and derived products such as wine and chocolate. Prospective studies have shown an inverse correlation between dietary flavonoid intake and mortality from coronary heart disease [11]. Several studies using various animal models provide support for the observed protective effects of dietary flavonoids with respect to cardiovascular diseases [12]. Quercetin exerts systemic, coronary and pulmonary artery vasodilatation and antiaggregant effects in vitro [13], [14], [15], and reduces blood pressure, oxidative status and end-organ damage in animal models of hypertension [16].

We hypothesized that quercetin could be effective in reversing PAH. Therefore, we tested the efficacy of oral quercetin in a rat model of PAH generated by a single injection of the plant toxin monocrotaline. This model reproduces several key aspects of PAH, including elevated PAP, RVH, premature death, vascular remodeling, oxidative stress, endothelial dysfunction, and alteration in the BMPR2, K_V_ and 5-HT pathways.

## Methods

### Ethics statement

The investigation conforms with the Directive 2010/63/EU of the European Parliament and the procedures were approved by our institutional Ethical Committee (Comité de Experimentación Animal de la Universidad Complutense de Madrid). All efforts were made to minimize suffering. Animals were monitored daily and eventually sacrificed by deep anesthesia followed by decapitation before the predefined duration of the treatment if death, due to right heart failure, could be anticipated based on immobility and general state of the animal.

### Animals and treatments

**Figure 1:**
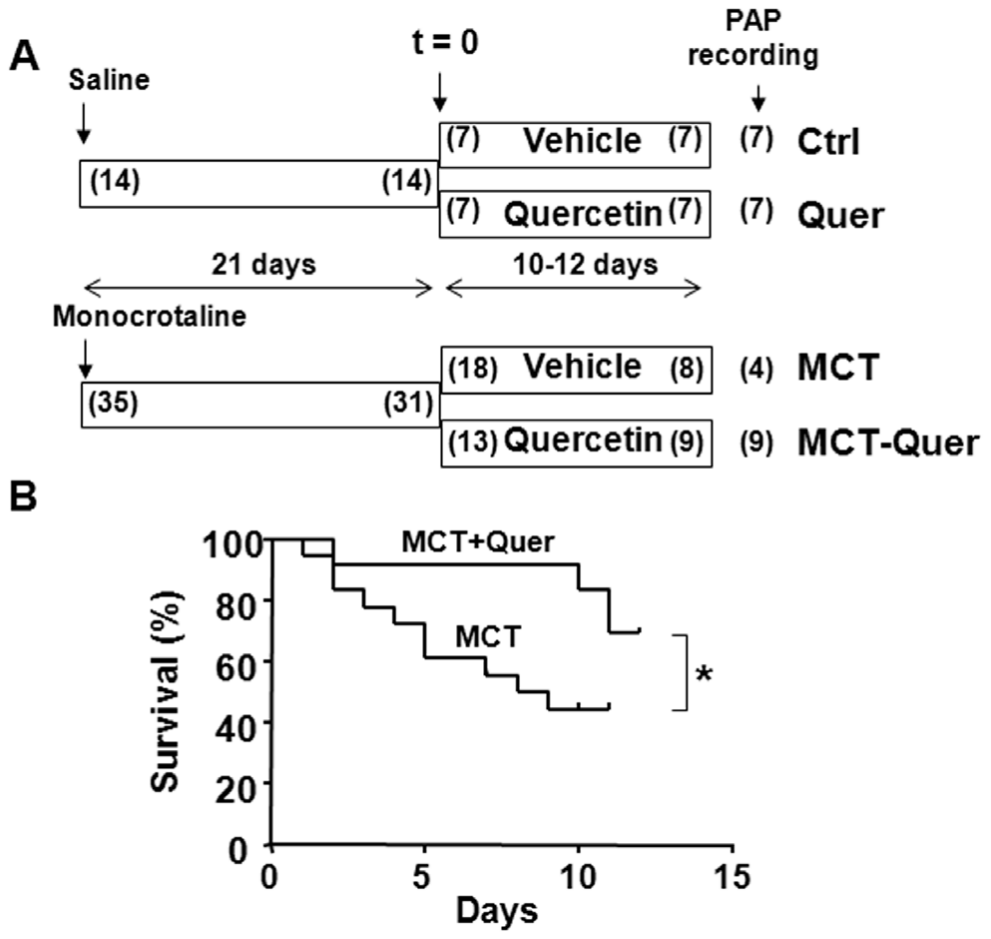
Quercetin increases survival. (A) Study protocol. Numbers in parenthesis indicate the number of rats which started and finished each period. The numbers at the far right indicate the number of rats in which pulmonary arterial pressure could be recorded; the missing animals in the monocrotaline (MCT) group died during the anesthesia or surgery. (B) Kaplan-Meier analysis of survival in rats treated with monocrotaline and monocrotaline plus quercetin (Quer). * indicates P<0.05.

The study protocol is shown in Figure 1. Male Wistar rats of 225–250 g of body weight (BW) from Harlan Iberica (Barcelona, Spain) were maintained in the general animal facility of Universidad Complutense (ANUC), five per cage, at a constant temperature (24±1°C), with a 12-hour light/dark cycle, on a standard chow and water *ad libitum*. Animals were randomly divided into a control and a pulmonary hypertensive group. Pulmonary hypertension was induced by a single intraperitoneal (i.p.) injection of 60 mg kg^−1^ monocrotaline (control animals were injected with saline). Twenty one days after monocrotaline injection, i.e. after the animals have developed marked increases in PAP, rats from both groups were further randomly assigned to vehicle or quercetin treatment (10 mg kg^−1^ once daily 9∶00–11∶00 AM, dissolved in 1 mL of 1% methylcellulose) by gastric gavage for an additional period of 11 days. This dose has widely demonstrated to be effective in systemic hypertension in rats [16] and the treatment period was chosen because in the 4–6^th^ week is the period of maximal worsening of the disease and higher mortality in this model. The study was run in three batches; all groups were followed in parallel in the first two and the two monocrotaline groups in the last one. The number of rats in each group was adjusted to reach a similar number of surviving rats in all groups at the end of the treatment based on the expected and actual mortality.

### Pressure measurements

Twenty-four h after the last administration of quercetin or vehicle rats were anaesthetized i.p. with 80 mg kg^−1^ ketamine plus 8 mg kg^−1^ xylazine and ventilated with room air (tidal volume 9 ml kg^−1^, 60 breaths min^−1^, positive end-expiratory pressure 2 cm H_2_O). Right ventricular systolic and diastolic pressure (RVSP, RVDP) and systolic, diastolic and mean pulmonary arterial pressures (SPAP, DPAP and MPAP) were then measured in open-chest rats with a pressure transducer via a catheter advanced through the right ventricle into the PA. It should be noted that the pressure and heart rate values obtained in anaesthetized open-chest rats may represent an underestimation of the values in closed-chest conscious animals. At the end of the recordings, hearts were excised and the right ventricle (RV) and the left ventricle plus septum (LV+S) were carefully dissected and weighed. The ratio RV/BW and the Fulton Index [RV/(LV+S)] were calculated to assess the right ventricular hypertrophy.

### Lung histology

The right lung was inflated *in situ* with formol saline through the right bronchus and embedded in paraffin. Lung sections were stained with haematoxylin and eosin and Masson trichrome techniques and examined by light microscopy, and elastin was visualized by its green autofluorescence. Small arteries (25–100 *µ*m outer diameter) were analysed in a blinded fashion and categorized as muscular, partially muscular or nonmuscular as previously described [17].

### Vascular reactivity

Intrapulmonary artery rings (2–3 mm long, ∼0.5–0.8 mm internal diameter) were dissected and mounted in Krebs solution bubbled with 21%O_2_ and 5% CO_2_ under 0.75 g of resting tension in organ chambers as previously described [18]. After equilibration, arterial rings from the four animal groups were firstly stimulated with KCl (80 mM). Thereafter, preparations were washed and after 45 min were contracted by 10^−7^M phenylephrine, and concentration–response curves to acetylcholine (10^−9^−3×10^−4^ M) were performed by cumulative addition. In different rings a concentration–response curves to serotonin (3×10^−8^−10^−4^ M) were performed by cumulative addition.

### K_V_ current recordings

For smooth muscle cells isolation, endothelium-denuded PA were cut into small segments (2×2 mm) and placed in Ca^2+^-free physiological salt solution containing (in mg/ml) 1 papain, 0.8 dithiothreitol and 0.7 albumin. Tissues were incubated in this solution at 4°C for 10 minutes and then agitated for 7 minutes at 37°C. Afterwards, tissues were washed in Ca^2+^-free physiological salt solution and disaggregated using a wide bore, smooth-tipped pipette. Cells were stored at 4°C and used within 8 h of isolation. Membrane currents were recorded using the whole-cell configuration of the patch clamp technique as previously described [19]. Membrane potential (Em) was measured under current clamp mode.

### Real time RT-PCR

Total RNA was isolated and purified from whole lung homogenates using RNeasy Mini kit (Qiagen, Hilden, Germany). Real-time PCR was performed using a Taqman system (Roche Diagnostics, Mannheim, Germany) in the Genomic Unit of Universidad Complutense de Madrid. Specific primers were designed for Kv1.5 (sense 5′-GGAAGAACAAGGCAACCAGA-3′, antisense 5′-AGCTGACCTTCCGTTGACC-3′), iNOS (sense 5′-TTGGAGTTCACCCAGTTGTG-3′, antisense 5′-ACATCGAAGCGGCCATAG-3′), eNOS (sense 5′-GGTATTTGATGCTCGGGACT-3′, antisense 5′-TGTGGTTACAGATGTAGGTGAACA-3′), caveolin-1 (sense 5′-GGGCATGAAGGCAGGTTAT −3′, antisense 5′-AGTGAGGACAGCAACCAACTC −3′), BMPR2 (sense 5′-CGGGCAGGATAAATCAGGA-3′, antisense5’-CAGGAAAGTAAATTCGGGTGA-3′), 5HT_2A_ (sense 5′-CTGCTCAATGTGTTTGTCTGG-3′, antisense 5′-GAACAACGTATATACCAGTGGATTGA-3′) and β-actin (sense 5′-GCCCTAGACTTCGAGCAAGA-3′, antisense 5′-TCAGGCAGCTCATAGCTCTTC-3′). Survivin, Kv 2.1, Kv 7.1 and Kv 7.5 expression was analyzed using commercial primers from Applied Biosystems. The data were corrected by the expression of β-actin and expressed as relative expression of target genes in control animals using the delta-delta Ct method (RQ).

### Western blotting analysis

Lung homogenates were run on a sodium dodecyl sulphate-polyacrilamide electrophoresis. Proteins were transferred to polyvinylidene difluoride membranes, incubated with primary antibodies against survivin, ERK1/2, p38, Akt, S6 or the phosphorylated forms of ERK1/2, p38, Akt and S6 240–244 (all from Cell Signalling Technology, Danvers, MA, except the anti-survivin and the anti-p-p38 antibodies from Santa Cruz, Dallas) and then with the secondary peroxidase conjugated antibodies. Antibody binding was detected by an ECL system (Amersham Pharmacia Biotech, Amersham, UK) and densitometric analysis was performed using Scion Image-Release Beta 4.02 software Samples were re-probed for expression of smooth muscle β-actin.

### PASMC and PA fibroblast proliferation

Pulmonary artery smooth muscle cells (PASMC) and fibroblasts were isolated from explants of PA from control and pulmonary hypertensive (21 days after monocrotaline treatment) rats and grown in Dulbecco's Modified Eagle Medium (DMEM) with 10% fetal calf serum. PASMC, seeded at 30.000 cell/ml (approximately 50% confluence) in 96 well plates, were growth arrested by an exposure for 24 h in 0.1% fetal calf serum (FCS) medium, and then exposed to quercetin or vehicle (0.1% DMSO) in DMEM containing 10% FCS for 24 or 48 h. Medium in all experiments using quercetin or vehicle was supplemented with ascorbic acid (30 µM) to minimize quercetin oxidation. Cell viability and proliferation were measured using the 3-[4,5-dimethylthiazol-2-yl]-2,5-diphenyltetrazolium bromide (MTT) [20] and the 5-bromo-2′-deoxyuridine (BrdU) assays (according to the manufacturer’s protocol, Roche Applied Science), respectively. Nuclear morphology was assessed after 24 or 48 h of treatment by incubating the cells with the membrane permeable DNA dye Hoechst 33258 (4 µg/mL) and the membrane impermeable DNA dye propidium iodide (20 µg/mL) for 40 min at 37°C followed by observation using a fluorescence microscope.

### Drugs and reagents

All drugs were from Sigma (Tres Cantos, Spain) except ketamine (Merial, Lyon, France) and xylazine (KVP Pharma und Veterinar-Produkte GmbH, Kiel, Germany).

### Statistical analysis

Results are expressed as means ± SEM of n measurements where n identifies the number of animals. Statistical analysis was performed by one-way ANOVA followed by Bonferroni’s *post hoc* test except for mortality which was assessed using a Kaplan-Meier analysis and for the PA muscularization study using a *χ*2 test. A value of *P<*0.05 was considered statistically significant.

## Results

### Survival, pulmonary artery pressure and right ventricular hypertrophy

All animals were healthy at baseline. Four out of 35 rats treated with monocrotaline died within the first three weeks, i.e. before randomization to quercetin-vehicle (Figure 1A). Only 8 of the 18 monocrotaline-treated rats assigned to vehicle treatment survived until the end of the experiment and PAP could only be recorded in 4 of them because the rest died during the anesthesia or surgery. These latter rats were not included in the mortality analysis but their organs and tissues were immediately dissected for further use. Four out of 13 monocrotaline treated rats died during the treatment with quercetin. The Kaplan-Meier analysis (Figure 1B) shows that quercetin produced a significant increase in survival.

**Figure 2:**
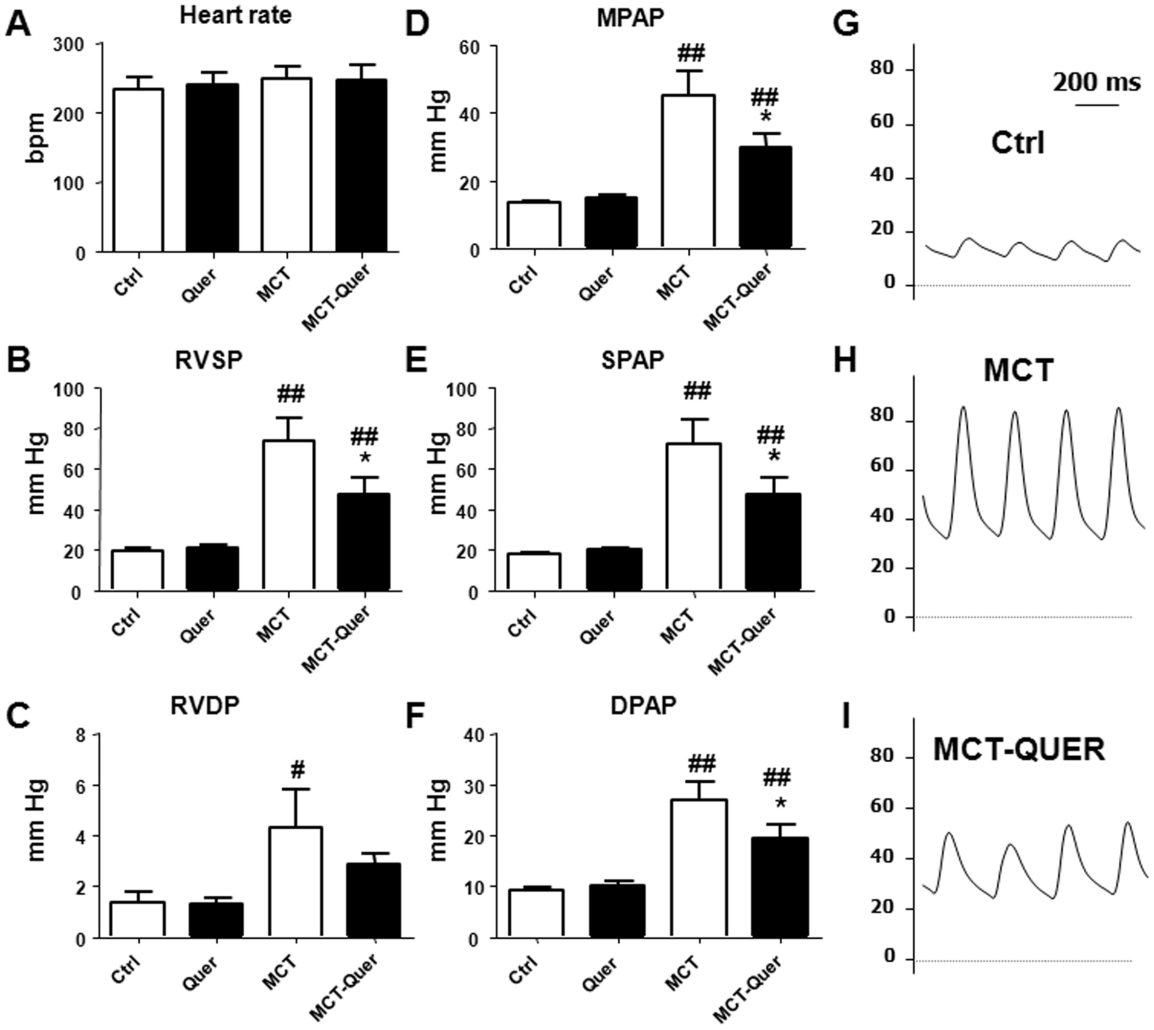
Quercetin reduces pulmonary artery pressure. Heart rate (A), right systolic (B) and diastolic (C) ventricular pressure, mean (D), systolic (E) and diastolic (F) pulmonary arterial pressure. Panels G, H, I show pulmonary artery pressure recordings in the control (Ctrl), monocrotaline (MCT) and monocrotaline plus quercetin (Quer) group, respectively. Results are means ± SEM of 4–9 animals, * indicates P<0.05 versus MCT and #P<0.05, ## P<0.01 versus Ctrl.

Monocrotaline produced significant increases in RVSP, RVDP, SPAP, DPAP and MPAP (Figure 2). Therefore, as expected, monocrotaline treated rats developed pulmonary hypertension, with MPAP values of 45.2±7.1 mmHg (vs. 13.6±0.6 mmHg in controls). An oral daily dose of quercetin starting three weeks after monocrotaline significantly reduced these parameters (except for RVDP) with MPAP values of 29.7±5.9 mmHg, which were still significantly above control values. The heart rate was similar in all experimental groups.

**Figure 3:**
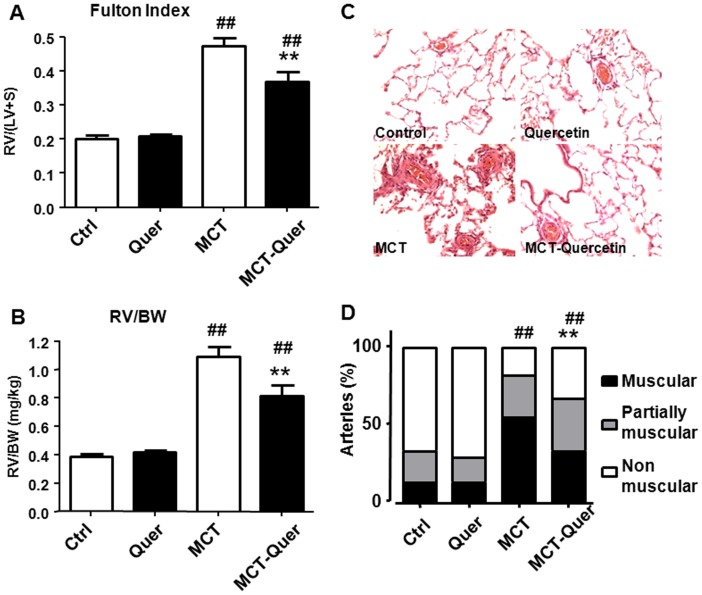
Quercetin reduces right ventricular hypertrophy and vascular remodeling. (A) Fulton index [RV/(LV+S) ratio]. (B) Right ventricular weight relative to body weight (RV/BW). (C) Representative images of lung histology. (D) Percentage of muscular, partially muscular and non-muscular arteries in different groups. For panels A and B each column represents the mean ± SEM of 7–9 animals. ** For panel D between 26 and 110 arteries (25–100 *µ*m) were analyzed in cross-sections of lungs (stained with haematoxylin and eosin) from at least four animals in each group. ** indicates P<0.01 versus monocrotaline (MCT) and ## indicates P<0.01 versus control (Ctrl).

Rats treated with monocrotaline developed a marked right ventricular hypertrophy as shown by the increased RV weight referred either to the LV+S weight or to BW (Figure 3A and B). Quercetin significantly reduced both parameters but again they were still significantly above controls.

### Histological changes

Small PA (25–100 *µ*m) observed in lung sections were classified as muscular, partially muscular and non-muscular arteries (Figures 3C and D). Monocrotaline increased the percentage of muscular arteries, with a corresponding decrease in partially muscular and non-muscular arteries. The treatment with quercetin resulted in a partial prevention of monocrotaline-induced vascular remodeling (P<0.01).

### K_V_ currents and membrane potential

**Figure 4:**
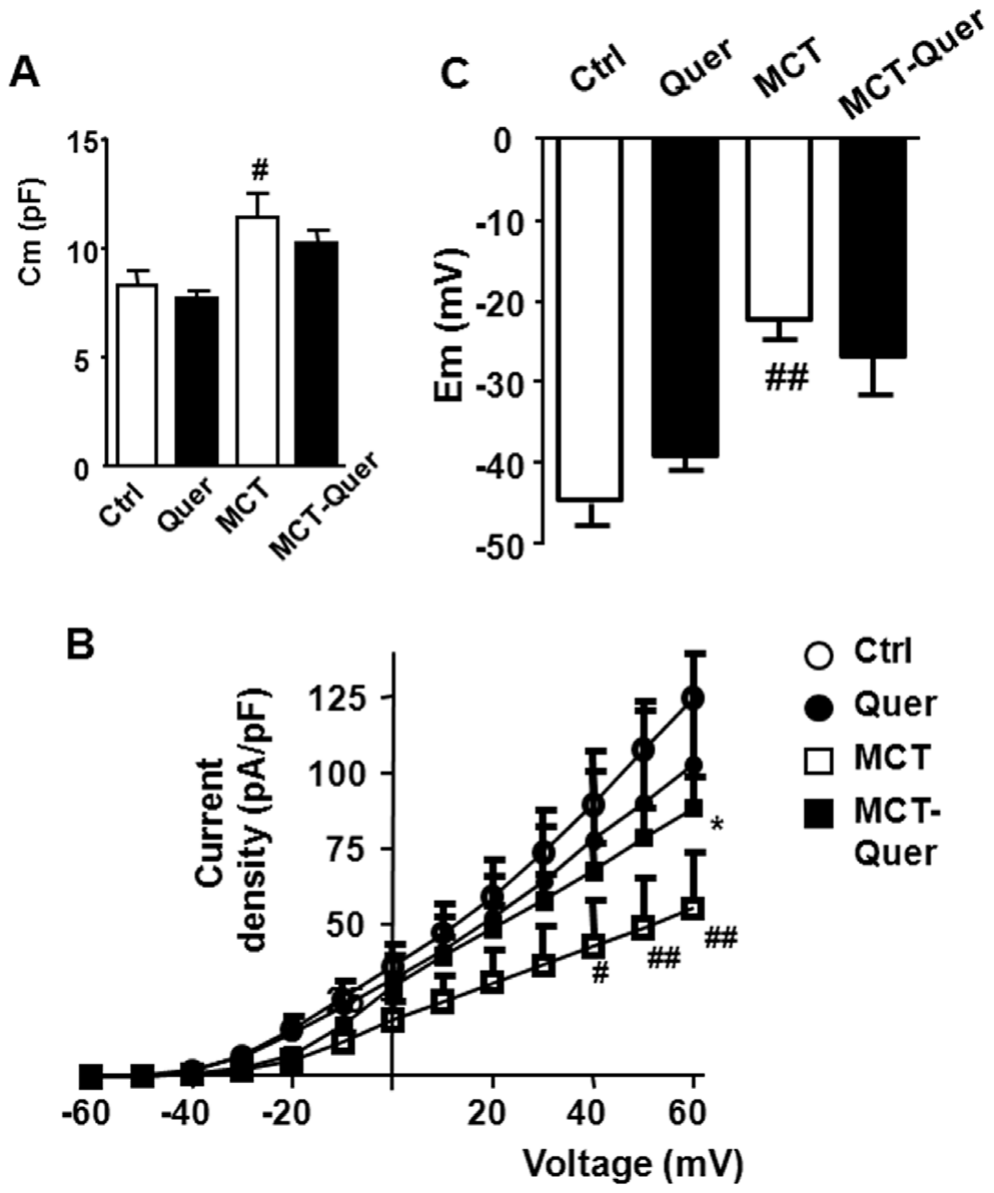
Quercetin prevents the inhibition of K_V_ currents. (A) Cell capacitance (Cm). (B) Current–voltage relationships measured at the end of the pulse. (C) Membrane potential (Em). * indicates P<0.05 versus monocrotaline (MCT). # and ## indicate P<0.05 and P<0.01 versus control (Ctrl). Each column or symbol represents the mean value ± SEM (n = 4–6).

In freshly isolated PASMC, membrane capacitance, an estimate of membrane surface, was significantly increased in monocrotaline treated rats. Quercetin was not able to restore this increase (Figure 4A). Monocrotaline also induced a significant decrease in the amplitude of the K_V_ currents and membrane depolarization. Quercetin significantly reversed the inhibition of the K_V_ currents but it had no significant effect on membrane potential (Figure 4B and 4C).

### Gene expression

**Figure 5:**
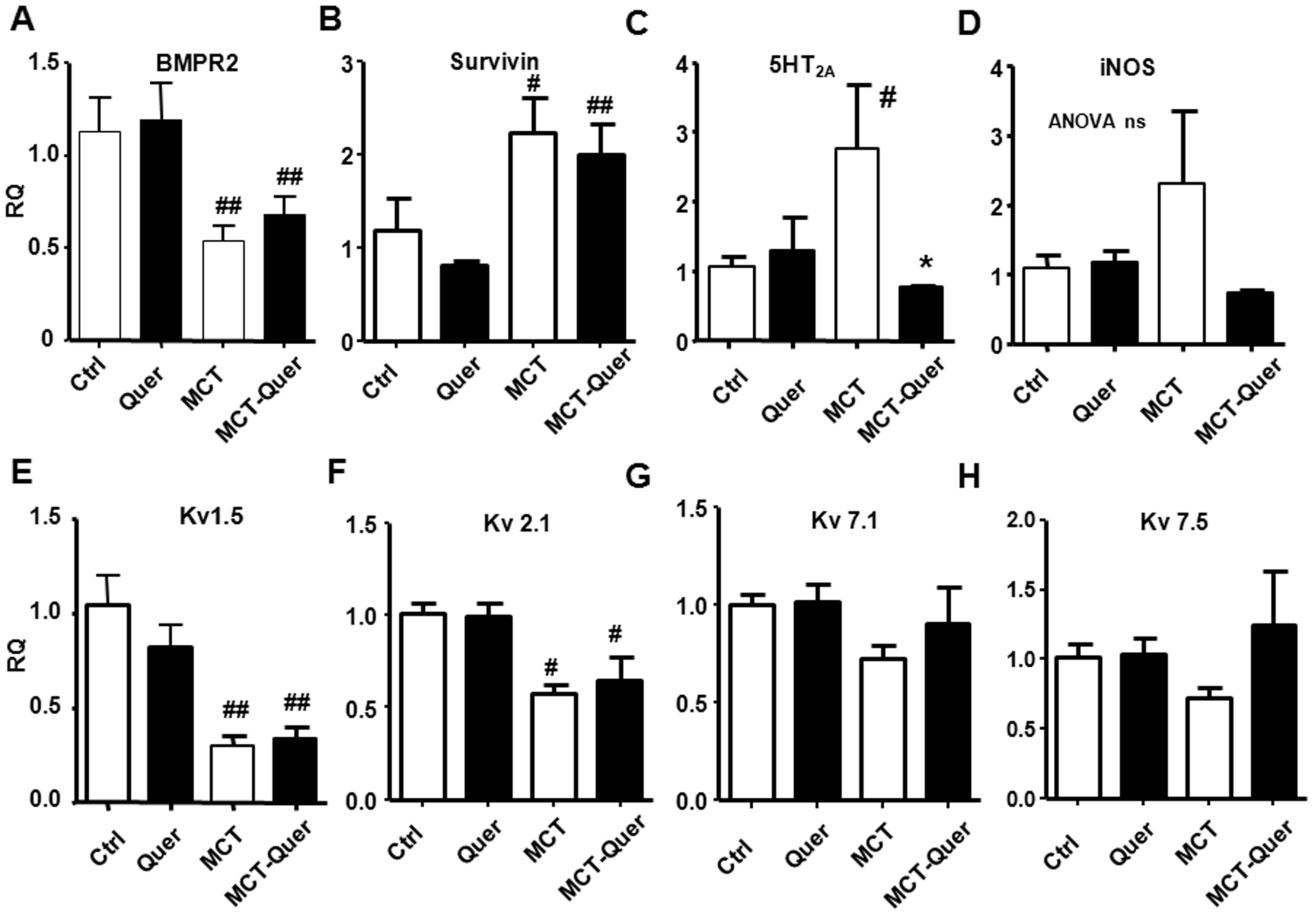
Changes in lung expression of (A) BMPRII, (B) Survivin, (C) 5HT_2A_, (D) iNOS, (E) Kv1.5, (F) Kv2.1, (G) Kv7.1 and (H) Kv7.5 mRNA by RT-PCR. Results are means ± SEM of 4–8 animals normalized by the expression of β-actin. * indicates P<0.05 versus monocrotaline (MCT), # and ## P<0.05 and P<0.01 versus control (Ctrl).

**Figure 6:**
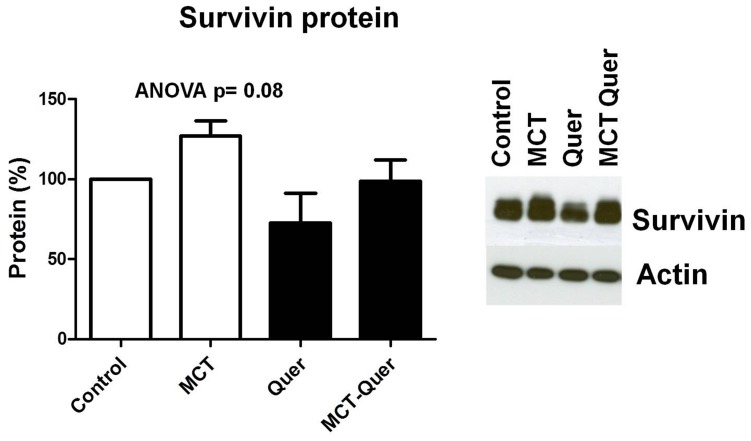
Changes in the expression of survivin at the level of protein. Lung homogenates were analyzed by Western blot. Results are means ± SEM of 5–8 animals. Protein levels were normalized by the expression of β-actin.

We examined the gene expression in whole lung homogenates of key proteins involved in PAH. BMPR2, Kv1.5 and Kv2.1 were strongly downregulated by monocrotaline (Figure 5). The difference in expression of these genes in the monocrotaline plus quercetin vs monocrotaline group was not statistically significant. The expression of survivin and 5HT_2A_ mRNA was significantly increased by monocrotaline, and quercetin was able to reverse the latter change. However, changes in survivin protein (Figure 6) were smaller and not significant among groups. The mRNA expression of iNOS in whole lung was highly variable in the monocrotaline group, despite a trend for increased expression in the monocrotaline group the ANOVA did not yield significant differences. Thus, we analyzed the mRNA expression of iNOS in homogenates from isolated pulmonary arteries. In this tissue, iNOS mRNA was significantly increased by monocrotaline (2.4±0.7 fold) and this effect was inhibited by quercetin (1.02±0.6). Despite a positive trend, the changes in expression of Kv7.1 and Kv7.5 in whole lung were not statistically different among groups.

### Vascular dysfunction

**Figure 7:**
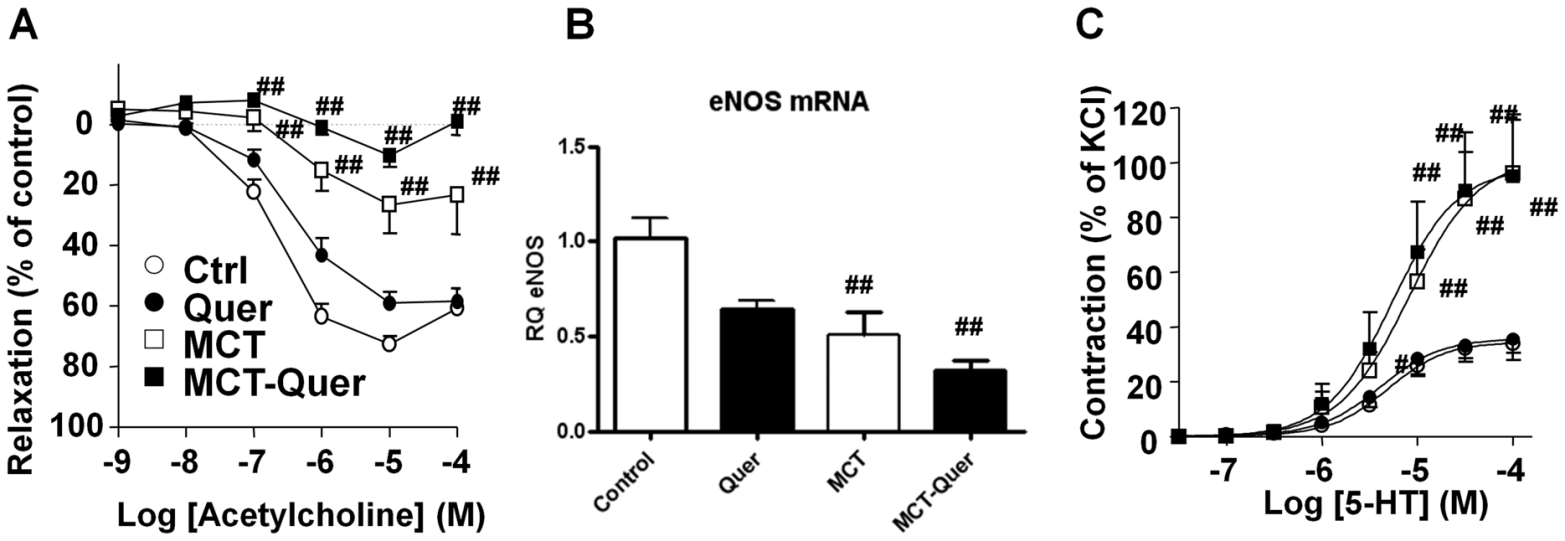
Quercetin (Quer) does not prevent vascular dysfunction. (A) Relaxant effects of pulmonary arteries to the endothelium-dependent vasodilator acetylcholine in pulmonary arteries stimulated with phenylephrine. (B) Expression of eNOS mRNA in pulmonary arteries homogenates analyzed by RT-PCR. (C) Contractile responses to 5-HT in pulmonary arteries expressed as a percent of a previous response to KCl. Results are means ± SEM of 4–9 experiments. Results are means ± SEM of 4–8 animals normalized by the expression of β-actin. * indicates P<0.05 versus monocrotaline (MCT), # and ## P<0.05 and P<0.01 versus control (Ctrl).

Monocrotaline significantly decreased the maximal relaxation evoked by acetylcholine in isolated rat pulmonary arteries (Figure 7A) and no changes were found in rats treated with quercetin. The endothelial dysfunction was accompanied by a reduction in the expression of eNOS which again was not reverted by quercetin (Figure 7B). Monocrotaline also produced a significant hyperresponsiveness of pulmonary arteries to 5-HT (E_max_ values of 102±3% of the response to KCl vs 35±1% in control) and quercetin again was not able to prevent it (98±1%) (Figure 7C).

### Expression of MAPK and Akt pathways

**Figure 8:**
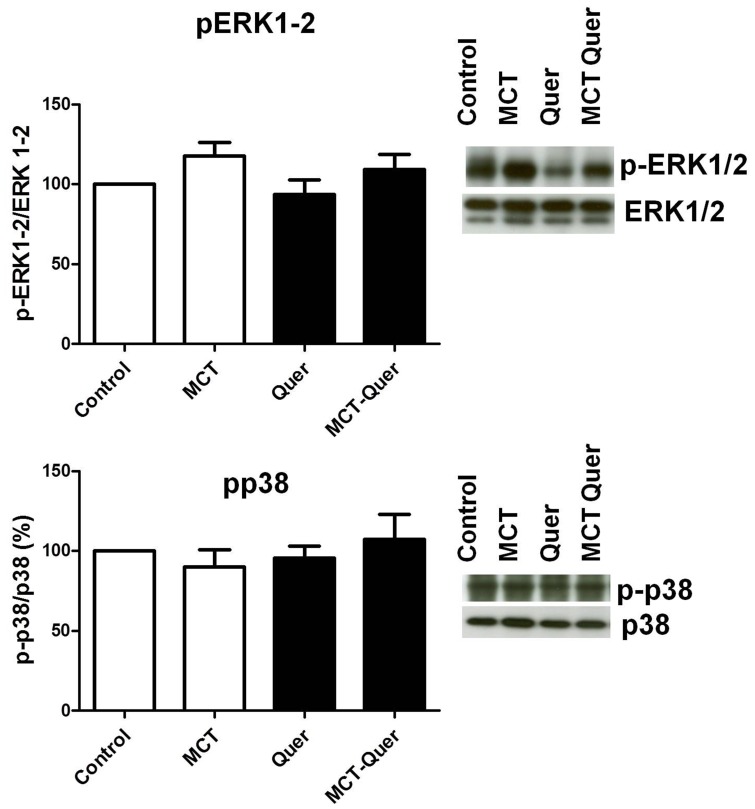
Lack of changes in the activation of MAPKs: ERK1/2 and p38MAPK. Lung homogenates were analyzed by Western blot and probed with the anti-phospho MAPK or the anti-total MAPK antibodies. Results expressed as phosphorylated forms normalized by the total protein, are means ± SEM of 5–8 animals.

**Figure 9:**
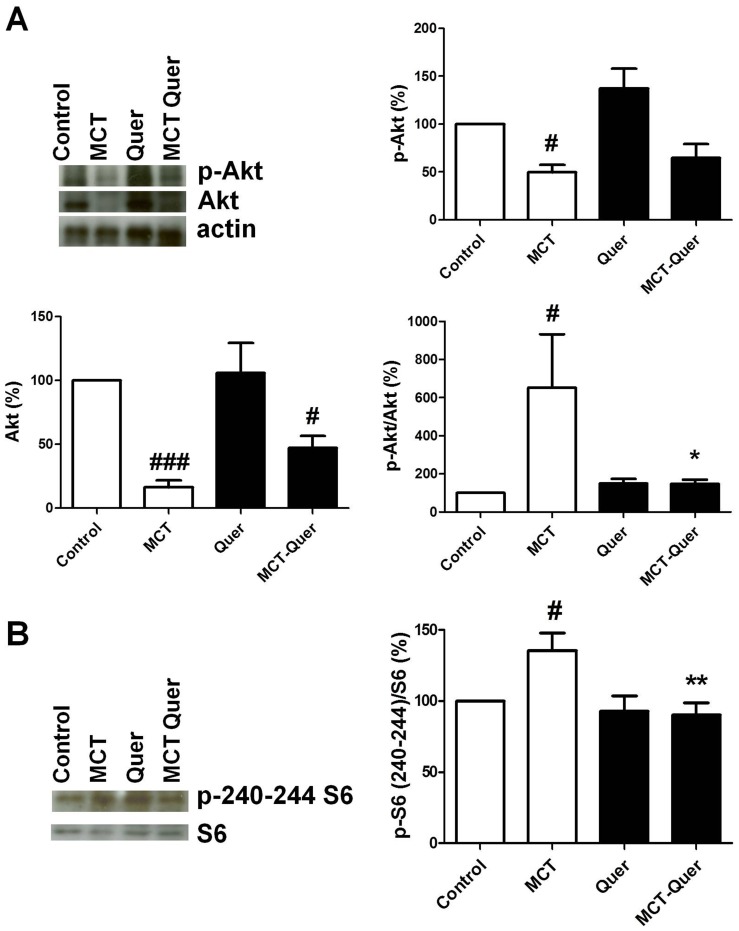
Changes in the PI3K/Akt/mTOR/S6 signaling pathway. Lung homogenates were analyzed by Western blot and probed with the anti-phospho Akt, the anti-total Akt or anti-β actin (A) or antiphospho S6 ribosomal protein (serines 240, 244) or total S6 (B) antibodies. Results expressed as phosphorylated forms normalized by the total protein, are means ± SEM of 5–8 animals. * indicates P<0.05 versus monocrotaline (MCT), #, ## indicate P<0.05, and P<0.0001 versus control (Ctrl).

Despite an apparent increase in phosphoERK1/2 in some monocrotaline-treated rats, on average, the phosphorylation of MAP kinases ERK1/2 and p38 was not significantly different (Figure 8A,B). Pulmonary hypertensive animals showed a marked increased phosphorylation of Akt (p-Akt to total-Akt ratio), and this effect was not evident in monocrotaline-quercetin treated animals (Figure 9A). However, it should be noted that the increased p-Akt/total-Akt ratio was accompanied by a strong downregulation of Akt. Phosphorylation of S6 at residues 240–244 was significantly increased by monocrotaline and this effect was prevented by quercetin (Figure 9B).

### Antiproliferative and pro-apoptotic effects *in vitro*

**Figure 10:**
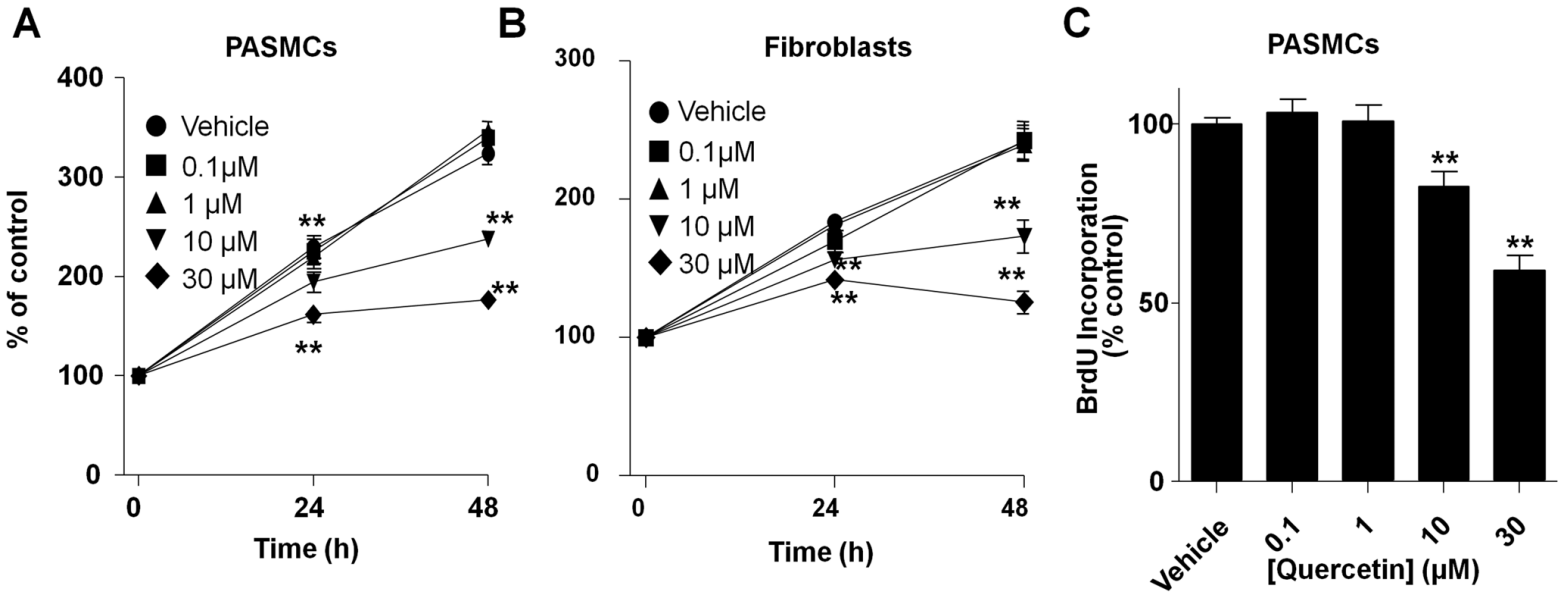
Quercetin decreases PASMC and fibroblast proliferation. (A) PASMC and (B) fibroblasts were isolated from monocrotaline-treated rats and grown in culture. Viable cells were estimated by the MTT test exposed to quercetin in culture for 24 or 48 h. (C) PASMC proliferation was analyzed by the BrdU incorporation after 24 h of treatment with increasing concentrations of quercetin. Results are means ± SEM of 3–4 experiments performed in triplicate. ** indicates P<0.01 versus vehicle (DMSO).

PASMC and fibroblasts from monocrotaline-treated rats cultured in the presence of 10% FCS grew at an exponential rate for 48 h. Addition of quercetin produced a concentration- and time-dependent decrease in the number of viable cells estimated by the MTT assay in both cell types (Figure 10A and 10B). We analyzed whether quercetin was reducing cell proliferation in PASMC from monocrotaline-treated rats by the analysis of BrdU incorporation. Quercetin produced significant inhibitory effects on BrdU incorporation at 24 h (Figure 10C).

**Figure 11:**
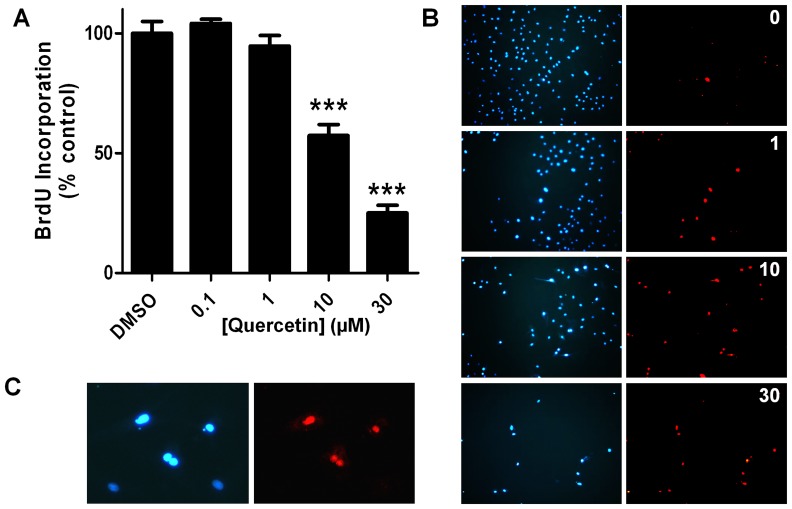
Quercetin decreases proliferation (A) and induces apoptosis (B) in cultured PASMC from control rats. (A) PASMC proliferation was analyzed by the BrdU incorporation after 24 h of treatment with increasing concentrations of quercetin. Results are means ± SEM of 4–5 experiments. (B) Photographs of PASMC after 48 h treatment with quercetin in culture stained with the membrane permeable blue dye Hoescht 33258 (left, viable and non-viable cells) and with the membrane impermeable red dye propidium iodide (right, non-viable cells). (C) Magnified photographs of cells treated with 10 µM quercetin showing three apoptotic cells (top and middle cells) with chromatin condensation and nuclear fragmentation and two healthy cells (bottom).

In PASMC from control rats, proliferation, measured by the MTT assay (not shown) or BrdU incorporation was also inhibited (Figure 11). It also strongly reduced the total number of cells (positive for the membrane permeable dye Hoescht 33258) and produced an increase in non viable cells (positive for the membrane impermeable dye propidium iodide, Figure 11). Non viable cells showed chromatin condensation and nuclear fragmentation characteristic of apoptotic cells.

## Discussion

PAH is characterized by increased pulmonary artery pressure as a result of increased pulmonary vascular resistance due to pulmonary vascular vasoconstriction, increased smooth muscle cell proliferation/apoptosis ratio and in situ thrombosis [3]. Quercetin has shown a number of vascular effects including vasodilator, antiplatelet, smooth muscle antiproliferative and pro-apoptotic effects in the systemic and coronary circulation. Herein we show that quercetin is also partially protective in a model of PAH by lowering PAP, RVH and vascular remodeling. In addition, *in vitro* quercetin exerted pulmonary vasodilator effects and inhibited cell proliferation in PASMCs.

PAH develops progressively and most PAH patients with earlier-stage disease such as New York Heart Association (NYHA) class I or II are asymptomatic and the condition is not identified until symptoms become severe [1]. Therefore, treatments for PAH must be effective once the symptoms have developed. Gao et al. have recently reported that a high dose of quercetin (10 fold the one we used) from day 2 to day 21 after monocrotaline and analyzing the results at day 41^st^, i.e. a preventive strategy, produced a reduction of PAP and RVH but they did not attempt to analyze the mechanisms involved [21]. We chose to start treatment with quercetin 21 days after monocrotaline. At this stage, the animals develop marked increases in PAP and the severity of the disease in our study was evidenced by 11% mortality. The final mortality in untreated animals was 56% (78% including animals which died during anesthesia or surgery). Moreover, surviving rats were in better hemodynamic conditions when treated by quercetin. As expected by the reduced right ventricular afterload, quercetin also decreased the right ventricular hypertrophy. The effectiveness of quercetin in this experiment with a very aggressive form of PAH suggests that it might prevent premature death in humans with PAH. It should be noted, however, that although all clinically useful drugs for PAH are effective in this animal model, not all drugs reversing monocrotaline-induced increased PAP are effective in clinical PAH [22].

Despite the increased survival and the hemodynamic, anatomical and histological improvement, most of the conventional markers of PAH such as membrane depolarization, endothelial dysfunction and downregulation of BMPR2 and K_V_1.5, were unaffected or modestly affected by quercetin. Endothelial dysfunction was accompanied by a reduced the expression of eNOS. This is consistent with previous studies showing that eNOS and other endothelial cell markers, such as caveolin-1 or CD31, are downregulated, suggesting that monocrotaline causes disruption of the integrity of the endothelial cells [23], [24]. Quercetin has demonstrated to improve endothelial function in several models of cardiovascular disease via decreased expression of NADPH oxidase subunits, restoring eNOS uncoupling, eNOS phosphorylation or scavenging superoxide [25]. However, quercetin was unable to restore eNOS downregulation which appears to be a major contributor to endothelial dysfunction in this model. In fact, a trend for reduced eNOS expression was found in quercetin-treated animals which is in agreement with *in vitro* data in human umbilical endothelial cells or in spontaneously hypertensive rats [26], [27]. Notably, quercetin inhibited the decrease in K_V_ currents and the overexpression of 5-HT_2A_ and iNOS induced by monocrotaline. Downregulated 5-HT_2A_ receptors might contribute to reduce arterial remodeling [28] but it was not sufficient to decrease the vascular hyperresponsiveness to 5-HT. This later effect is probably related to changes in reactive oxygen species and cyclooxygenase activity as observed in other models of PAH as it can be normalized by cyclooxygenase inhibitors and antioxidants [29], [30], [31]. The fact that quercetin inhibits the changes in specific genes but does not affect other genes may reflect that changes in gene expression in this model of PAH operate through quercetin-sensitive and -insensitive signaling pathways.

Proliferation of PAMSC leading to increased muscularization of small PA and reduced vascular lumen is considered the hallmark of the most advanced and aggressive forms of PAH. Quercetin reduced the number of muscular arteries, an effect that is likely to contribute to the decrease in PAP. In order to confirm the *in vivo* findings, we tested the effects of quercetin on PASMC *in vitro*. We found that quercetin produced a concentration-dependent decrease in cell number of PASMC and fibroblasts, related to the inhibition of proliferation, as measured by the BrdU incorporation. However, quercetin also induces apoptosis in vascular smooth muscle [20] which may also marginally contribute to reduce cell viability in these *in vitro* experiments.

A large number of cell signaling pathways may be affected by quercetin. In fact, quercetin is a broad-spectrum protein kinase inhibitor being a competitive and reversible ligand for the ATP binding site [32]. However, few studies have confirmed it in its *in vivo* effects. Therefore, we analyzed in the rat lungs from the *in vivo* study the signaling pathways reported to be involved in the pathophysiology of PAH which could be potentially targeted by quercetin. These include those activated by platelet derived growth factor (PDGF) and transforming growth factor-β (TGF-β): MAPKs, the PI3K- Akt-mTor pathway, and the antiapoptotic protein survivin.

Quercetin is a canonical pan-inhibitor of MAPKs and the TGF-β pathway [33] and previous studies on the effects of quercetin on vascular smooth muscle cell viability, migration, and proliferation have related their findings to interactions with MAPKs [20], [34], [35]. MAPKs (e.g. ERK1/2, p38MAPK and JNK) play a key role in cell proliferation and this axis has been suggested as a potential therapeutic target in PAH [6]. However, their role in PAH induced by monocrotaline is controversial. In fact, increased [36], unchanged [37] or decreased [38] phosphorylation of p38MAPK and unchanged [36], [37] or increased [6] phosphorylation of ERK1/2 have been reported. We found no significant changes among groups in p38MAPK and ERK1/2 phosphorylation suggesting that MAPKs play little role in the PAH and in the effects of quercetin in this model.

Another potential pathway for PAMSC muscularization in PAH is via overexpression of the antiapoptotic protein survivin. In fact, genetic targeting of survivin has been shown to reduce PAH in the monocrotaline-treated rats [39]. We found that survivin mRNA was significantly overexpressed in the lungs of monocrotaline-treated rats but the protein increase did not achieve statistical significance. This difference might be due to post-transductional changes. Interestingly, quercetin has been shown to induce survivin downregulation in several cancer cells, via decreasing the survivin promoter activity [40]. Herein we show that chronic quercetin had no clear effect on survivin expression, suggesting that *in vivo* the downregulation of survivin is not a major mechanism for the antiproliferative effect. This is consistent with the high concentrations (50 µM) of quercetin required to downregulate survivin [40].

The intracellular signaling pathway involving the PI3K/Akt/mTOR/S6 plays an important role in cell proliferation in PAH and it is thought to be a promising therapeutic target for this syndrome [41], [42], [43]. Quercetin may interfere with the activity of both PI3K and mTOR [44], [45]. We found that Akt phosphorylation was increased by monocrotaline which was associated by a strong downregulation of Akt expression. As previously reported in other cell types [45], [46], the Akt phosphorylation was diminished by quercetin, suggesting that inhibition of this pathway may be involved in the decreased PA muscularization. This is consistent with the high potency of quercetin to inhibit PI3K (K_D_ at submicromolar concentrations) [32]. Furthermore, we also found that phosphorylation of S6, the downstream target of mTOR, was higher in the group treated with monocrotaline and this increase was inhibited by quercetin.

Quercetin is also a well-known vasodilator [13], [18]. We also found that quercetin was similarly effective in isolated PA both from control and monocrotaline-treated rats stimulated by the thromboxane A_2_ mimetic U46619 (−logIC_50_ of 5.3 and 5.1, respectively, not shown). Thus, this vasodilator effect may be an additional contributor to the reduction in PA pressure in this model.

The pharmacokinetics of quercetin are complex. It is not present in the blood in its free form but circulates conjugated with glucuronide which is released in the tissues after deconjugation [47]. Because quercetin was not administered the day of the end-point measurements, it is likely that the effects observed reflect the chronic rather than the acute actions of quercetin and the reduction in PA pressure may be underestimated.

In conclusion, quercetin is partially protective in this rat model of PAH. It delayed mortality by lowering PAP, RVH and vascular remodeling. Quercetin exerted effective vasodilator effects in isolated PA, inhibited cell proliferation and induced apoptosis in PASMCs. These effects were associated with decreased 5-HT_2A_ receptor expression and Akt and S6 phosphorylation and partially restored Kv currents. Therefore, quercetin could be useful in the treatment of PAH.
